# A 3D-Printable, Low-Cost Obturator for Less Invasive Gynecologic Brachytherapy

**DOI:** 10.7759/cureus.41162

**Published:** 2023-06-29

**Authors:** Sanika Rane, Alexander Hanania, Elisa Arango, Krithika Kumar, Lauren Payne, Susannah Dittmar, Gaurav Gomber, Vincent Ugarte, Michelle Ludwig

**Affiliations:** 1 Radiation Oncology, Baylor College of Medicine, Houston, USA; 2 Radiation Oncology, Dell Medical School, The University of Texas at Austin, Austin, USA

**Keywords:** high dose rate interstitial brachytherapy, treatment of cervical cancer, gynecologic and reproductive tumors/malignancies, three-dimensional (3d) printing, dosimetry plan, medical device technology, intracavitary therapy brachytherapy, gynecologic cancers, invasive cervical cancer, prototype design

## Abstract

The purpose of this report is to design, develop, and evaluate a cost-effective applicator for interstitial brachytherapy (ISBT) to minimize patient morbidity and facilitate access to curative radiation treatment for gynecologic cancers, especially in low-resource settings.

A computer-aided design and prototype were developed of a proposed applicator that incorporates 44 slotted channels to gently guide needles, with or without a tandem, through the vaginal canal, effectively eliminating the need for transcutaneous needle insertions typically employed during ISBT of advanced gynecologic cancer and thus reducing the risk of vaginal laceration and bladder or rectal injury. The tested prototype was developed using AutoCAD software (Autodesk, San Francisco, CA) and 3D printed in Accura Xtreme Gray material using stereolithography. Small-scale iterative tests using a gelatin phantom were conducted on this prototype to confirm the efficacy of the applicator through inter-operator usability, needle stability, and needle arrangement.

A promising prototype was developed aimed at addressing key issues with traditional perineum-based templates to facilitate ISBT, including being able to cover bulky tumors with parametrial extension reliably, decrease the risk of tissue or organ injury, and treat women with a prior hysterectomy. Results of preclinical testing demonstrated that the applicator met its purpose, suggesting that it may facilitate ISBT without the morbidity typically associated with the procedure, especially by addressing concerns associated with implementing the procedure in low-resource settings.

The applicator shows substantial promise in the treatment of advanced gynecologic cancer. While further testing remains necessary to confirm its translatability to the clinical setting, the applicator appears capable of meeting its design objectives, representing its potential for improving upon current methods.

## Introduction

Cervical cancer is the fourth most common cancer in women with an estimated 570,000 women diagnosed worldwide in 2018 [1]. Many of these cases can progress to later stages, especially in low- to middle-income countries (LMICs) [2]. These populations often have limited access to medical care, leading to a disproportionate burden of cervical cancer in these settings with 85% of incident cases and deaths occurring in LMICs like Malawi, India, and Brazil [3-5]. Unfortunately, late-stage cervical cancer has only a 58% five-year survival rate with regional involvement of the cervix and uterus and only an 18% five-year survival rate with distant involvement of the bladder and rectum, and these statistics only represent cases in the United States after treatment [6]. Globally, these rates are difficult to quantify given the lack of access to treatment, likely contributing to over 250,000 deaths from cervical cancer each year in LMICs alone [7].

Late-stage cervical cancer is usually characterized by geometrically challenging tumors that have extended laterally within the parametrium and even metastasized to surrounding areas. Interstitial brachytherapy (ISBT) is a highly effective, curative radiotherapy treatment, especially for cases involving such tumors. Current methods of brachytherapy treatment often rely on transcutaneous needles that are pushed through healthy skin and tissue to deliver radiation, so these needles are associated with pronounced morbidity, sometimes even making it difficult to treat late-stage tumors [8]. During needle placement, standard templates, applicators, and obturators, such as the Syed-Neblett template (Best Medical International, Springfield, Virginia), are also used to provide healthcare professionals with an approximate guide to the anatomy of the vaginal canal. Although these devices help orient them to some degree, healthcare professionals must still rely on ultrasound imaging to visualize the placement of the needles at the risk of damaging surrounding structures such as the bladder and rectum. Even with conscientious placement, interstitial needles that enter through the perineal tissue still contribute to significant pain and bleeding in patients, especially during removal [9].

Due to these concerns, ISBT can be a time-intensive procedure requiring a significant amount of skill to place needles accurately, and even then, the needles still contribute significantly to patient morbidity. To address these issues, hybrid interstitial intracavitary applicators are being more frequently used to administer brachytherapy, especially given their success in treating advanced tumors compared to traditional templates. However, the use of these applicators can cost upwards of $40,000 in the United States (further discussed in Methods). And while these applicators are invaluable in treating cervical cancer, in LMICs, many hospitals lack the infrastructure - from funding to equipment to qualified healthcare providers - to accommodate such a technical procedure for all of their patients, so unfortunately, access to ISBT is limited where it is needed the most.

This article was previously presented as a meeting abstract at the 2021 American Brachytherapy Society Annual Meeting on June 25, 2021.

Related work

A review of the literature revealed several projects that have attempted to combat similar issues by specifically creating customized 3D-printed applicators. Custom templates created using computed tomography (CT) images and MRI scans have been proposed to optimize designs to patient geometry, allowing healthcare professionals to place needles with extreme accuracy. Sethi et al. developed 3D-printed custom applicators for a combination of vaginal and ISBT, successfully placing needles under transrectal ultrasound (TRUS) guidance [10]. Laan et al. similarly developed 3D-printed custom applicators but incorporated custom vaginal topography and source channels for guidance instead [9].

These customizable applicators were found to be highly effective, but they required significant resources to develop. First, high-resolution imaging had to be done to determine the individual anatomy of each patient. Then, each patient’s anatomy, tumor location, and tumor size had to be studied to create a 3D template designed specifically for their case. And once the applicator had been made, it was most effective only in the first treatment session as tumor shrinkage caused a poor fit in subsequent sessions [9,10]. Though designing and implementing custom applicators significantly reduces the morbidity associated with the treatment of complex cervical cancer cases, this solution unfortunately appears impractical, especially in low-resource settings, like LMIC, where time, financial support, and equipment are limited.

Device objective

To improve upon current methods of brachytherapy treatment for cervical cancer by developing a universal obturator, the device should be compatible with most brachytherapy equipment, be affordable to ensure accessibility in low-resource settings, and should make brachytherapy easier to perform by minimizing both the time spent in the operating room and the skill required to accurately place interstitial needles. Moreover, by meeting these objectives, this device could reduce patient morbidity by decreasing the risk of complications typically associated with the procedure. This would allow more hospitals to be able to utilize ISBT, more healthcare professionals to be able to perform the procedure, and thus increasing patient access to brachytherapy globally.

The Universally Friendly Obturator, or UFO, was developed with these criteria in mind to address common issues with ISBT and increase accessibility to comprehensive treatment of complex cervical cancer cases, especially in low-resource settings. To specifically address issues identified with transcutaneous needles, the UFO incorporates 44 angled channels that gently guide needles throughout the vaginal canal to the cervix, allowing needles to reach up to a 9-cm tumor for treatment and effectively eliminating the need for transcutaneous placement. It should be noted that the UFO combines features of the traditional Syed-Neblett template with a vaginal obturator, so in this paper, the UFO will simply be referred to as an "obturator," rather than an applicator or a template, out of convenience.

In this paper, the process of prototyping the UFO, key features of its design, analysis of its efficacy via dose distribution modeling, and future implementation considerations will be discussed.

## Technical report

Materials and methods

Prototyping

**Figure 1:**
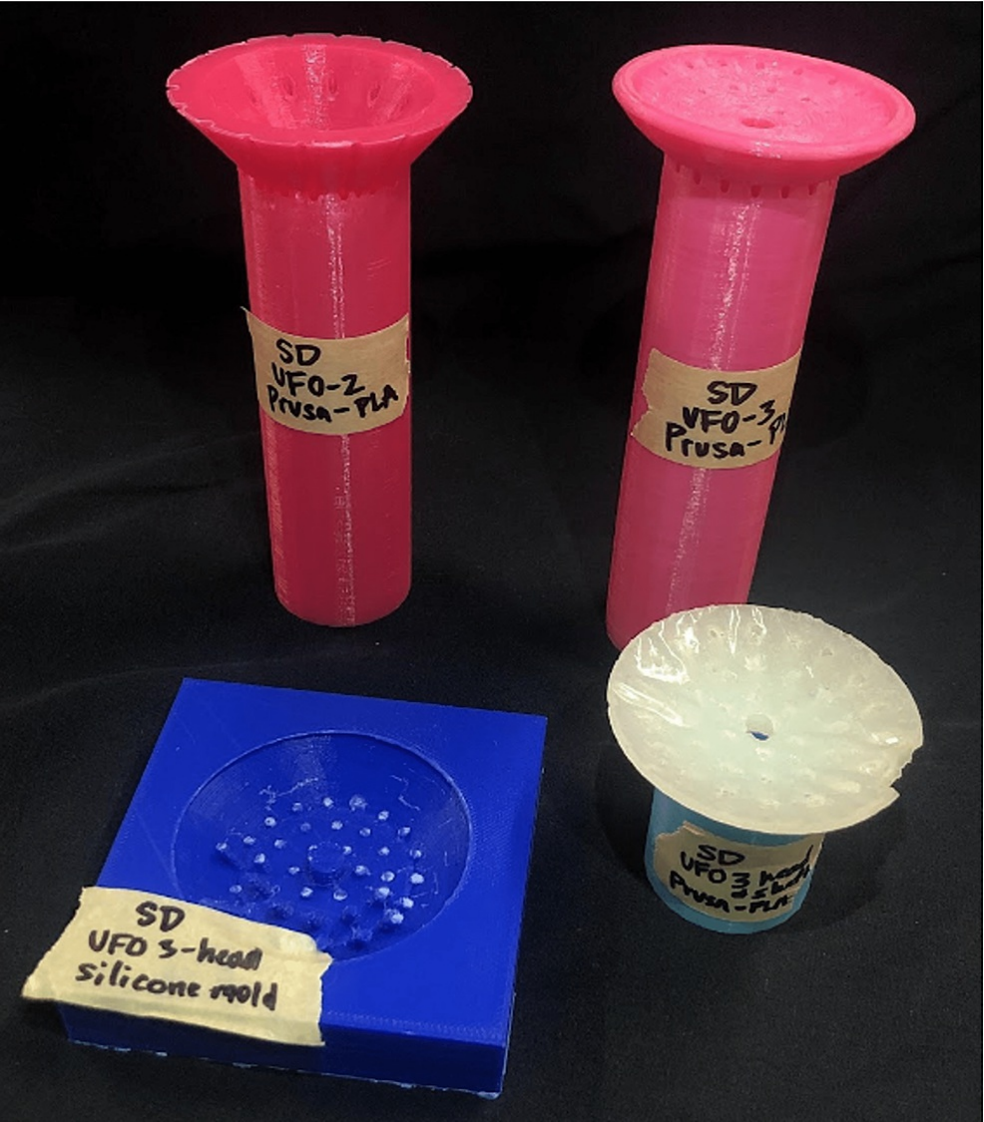
Early printed iterations of the device UFO: Universally Friendly Obturator.

**Figure 2:**
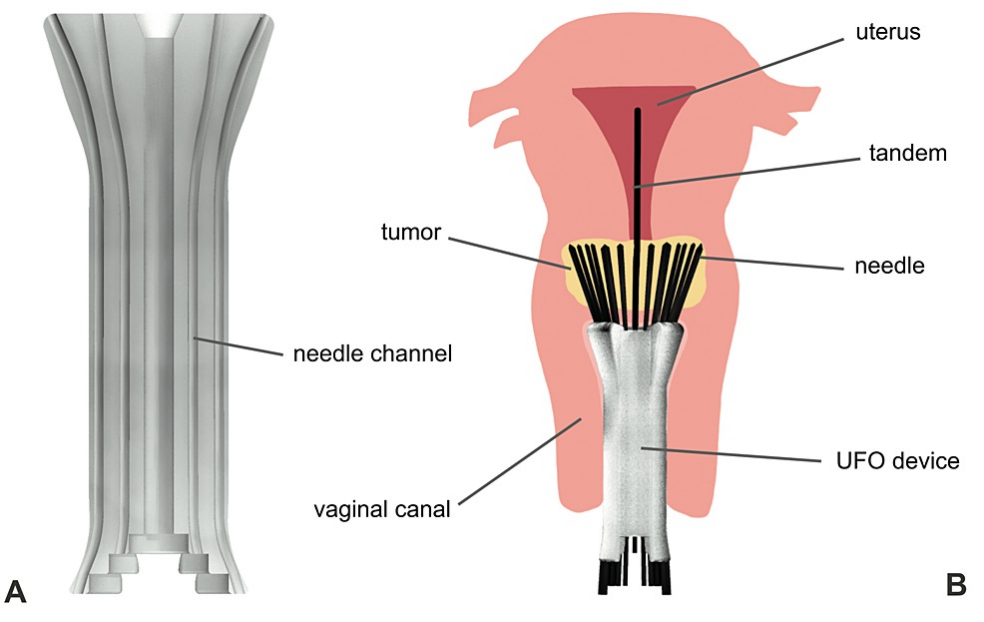
Overview of finalized device design (A) Coronal slice of Universally Friendly Obturator (UFO) device showing internal channels. (B) Diagram of the device being used to treat a tumor.

**Figure 3:**
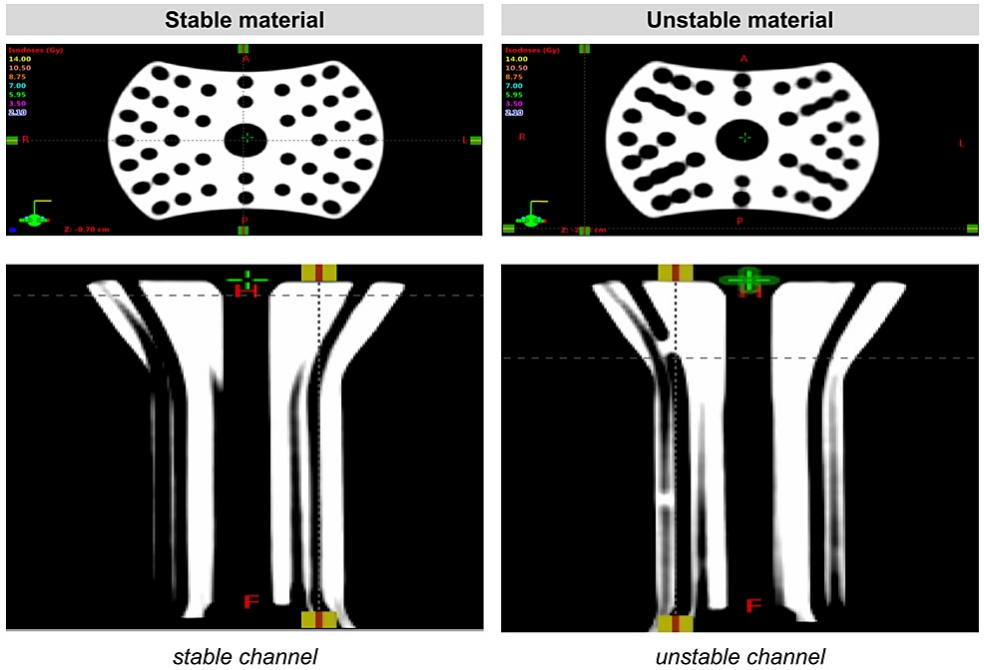
Comparing CT scans with needle trajectories of UFO device iterations printed in stable material to unstable material UFO: Universally Friendly Obturator.

After several rounds of brainstorming designs, the UFO was developed. The design involved an obturator that fits comfortably inside the vaginal canal, protecting surrounding tissue by confining needles within its internal channels until they reach their intended site, the tumor, to deliver radiation. Early prototypes were produced using AutoCAD software (Autodesk, San Francisco, CA) and 3D-printed in polylactic acid (PLA) material on Prusa i3 MK3S (Prusa Research, Prague, Czech Republic) and Ultimaker 3 series (Dynamism, Chicago, IL) extrusion printers. The first viable iterations (Figure 1) incorporated a hollow cylinder connected to a round head with shorter channels to direct needles into the proper equidistant arrangement of concentric rings. With proof of concept established, design alterations and considerations for manufacturing could be determined. In subsequent prototypes, the channels were extended to run through the entire length of the device, the shape was narrowed from its original circular form to an oblong shape, a stair-step interface was added on the user end, and the device dimensions were optimized (Figure 2). With design improvements, manufacturing methods, especially molding, were concurrently explored using low-fidelity models. Printing stability was assessed by manually inserting needles/tandem into the prototypes and confirming trajectories with CT scans with materials resulting in blocked channels and/or channel width fluctuations being classified as unstable (Figure 3).

Throughout the prototyping process, additional minor alterations were made to the design. Curves and edges in the shape were smoothened, and the angle of the channels exiting the device was increased slightly to widen the spread of the needles.

Current Design

**Figure 4:**
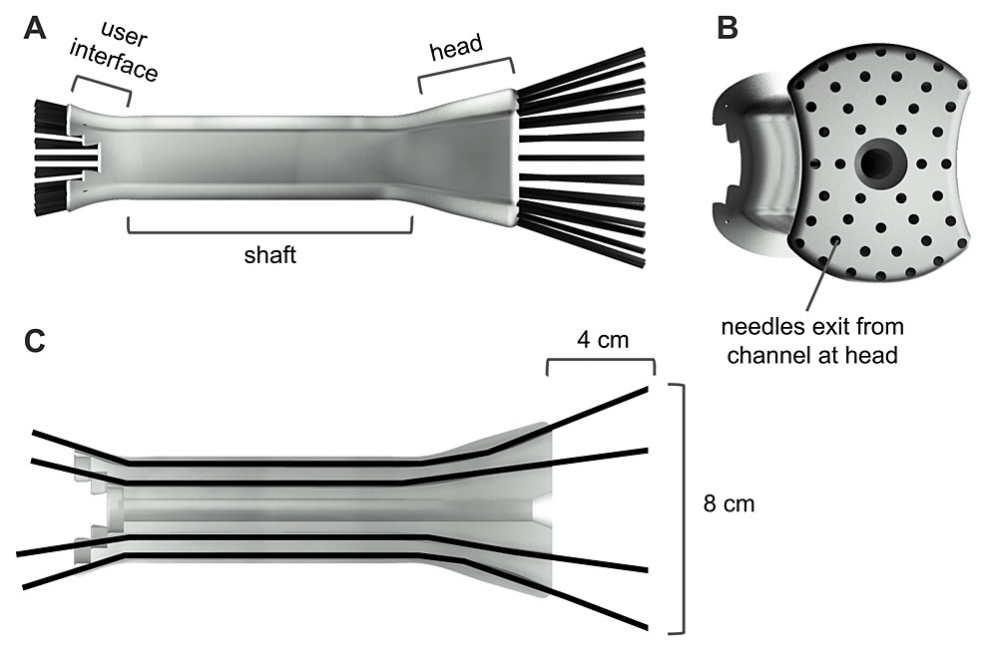
Components of finalized device design (A) Labeled components of the device (seen with needles inserted), (B) device head, and (C) coronal slice of the device demonstrating the length and width of possible needle trajectory.

The current UFO can be segmented into three parts: a user interface, a shaft, and a head (Figure 4). The user interface, where the needles enter the channels, is 4.6 centimeters wide and 1.5 centimeters long. As it becomes the shaft, it compresses to 3.5 centimeters wide. The shaft continues for 7.5 centimeters or 9.5 centimeters, depending on the desired length, before widening to become the head. The head, where the needles exit the channels, is 6 centimeters wide at its widest point and 4 centimeters long.

**Figure 5:**
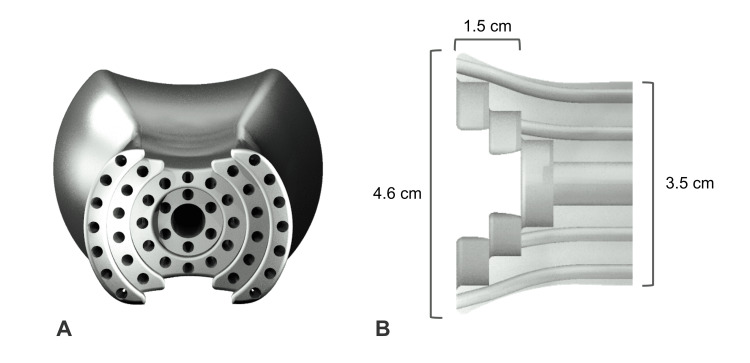
Stair-step design at the user interface of the device (A) User interface at the end of the device. (B) Cropped coronal slice demonstrating the length and width of the user interface.

The user interface employs a stair-step design, allowing healthcare professionals to easily differentiate between the concentric levels of angled channels (Figure 5). Differentiation at this level is necessary for healthcare professionals to be able to match each needle and its target with its appropriate dose.

The shaft serves as a bridge, connecting the user interface and the head. It contains 44 channels that guide needles throughout the vaginal canal to the cervix. As the shaft widens to form the head, the needle channels arc outwards before straightening out to a wider angle. The bend of the channels at the head precisely controls the spread of the needles, allowing them to reach the outer edges of a tumor.

The UFO is available in several options based on patient anatomy. There are two lengths available, 13 centimeters and 15 centimeters, depending on the length of the patient’s vaginal canal. There are also two options for the central channel, one that fits a tandem and one that fits a needle, depending on whether the patient has had a complete hysterectomy or has an intact uterus. By offering an option that fits a needle instead, the UFO is able to offer comprehensive radiation to these patients.

**Figure 6:**
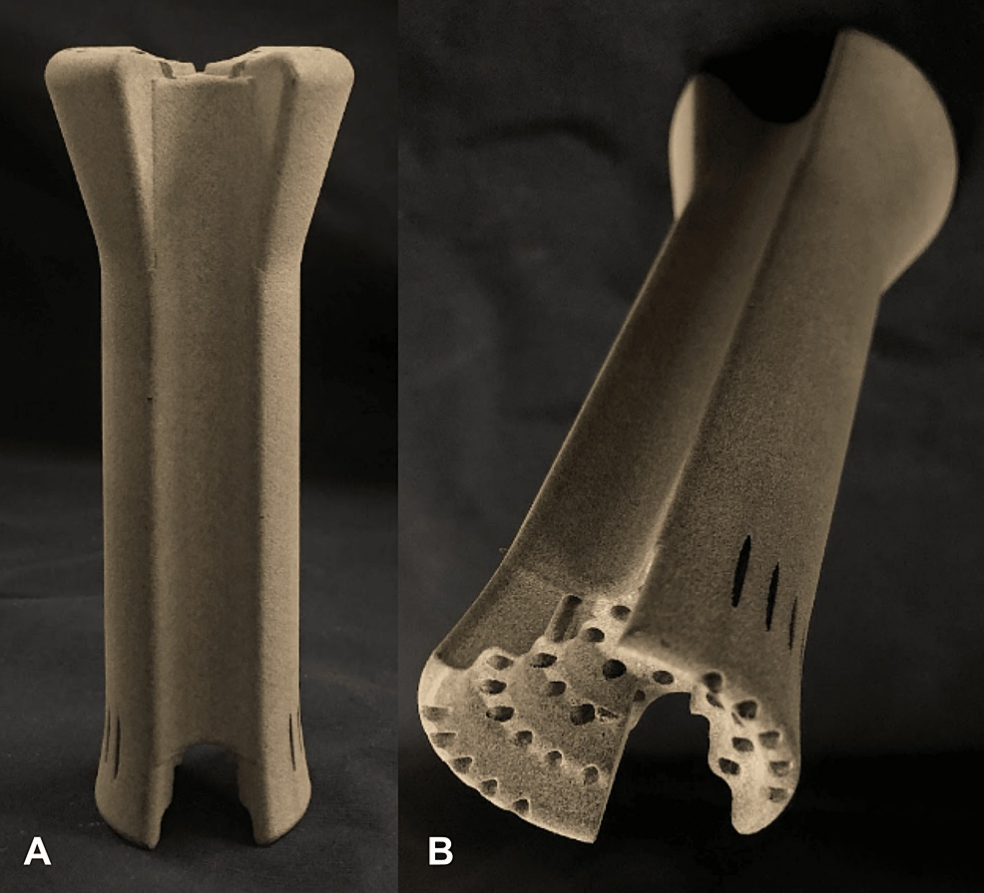
Current device design printed in Accura Xtreme Gray using stereolithography (A) Printed prototype. (B) Stair-step design of the user interface at the end of the device.

The prototype of the UFO was 3D-printed in Accura Xtreme Gray, an acrylonitrile butadiene styrene (ABS)-like material, using stereolithography (Figure 6). Accura Xtreme Gray was selected due to its biocompatibility, printer compatibility, and ability to be sterilized using ethylene oxide gas sterilization.

Testing of the Device

Iterative small-scale tests were conducted throughout this process of optimization. Formalized larger-scale testing was conducted after the completion of major design alterations. Tests to assess usability, needle stability, and needle arrangement were largely conducted using a cervical phantom made of gelatin and Metamucil, as described by Madsen et al. [11]. Usability was assessed following needle insertion into the phantom.

When planning for brachytherapy treatment, medical physicists work with radiation oncologists to determine the optimal needle arrangement necessary to reach the full extent of the tumor. This theoretical planning, done prior to the actual procedure, is executed using high-dose radiation (HDR) software. The software depicts the obturator to be used and where the needles are to be placed using the device. Thus, needle arrangement with the UFO was assessed by both theoretical planning with HDR software and physical testing with the phantom. Needle stability was assessed by measuring the length of needle displacement following manual disturbance of the phantom with needles placed in the UFO.

Testing for Usability and Needle Arrangement

A physics quality assurance (QA) of the device allowed for the assessment of both printing material stability and usability of the device in HDR planning software, a gold standard routinely used to determine needle arrangement and dosing distribution to generate an individualized treatment plan based on a patient’s tumor burden. After the best material for printing the UFO was determined, this theoretical assessment was generated depicting the device in HDR planning software and retrospectively compared to the Syed-Neblett template currently used to treat patients.

**Figure 7:**
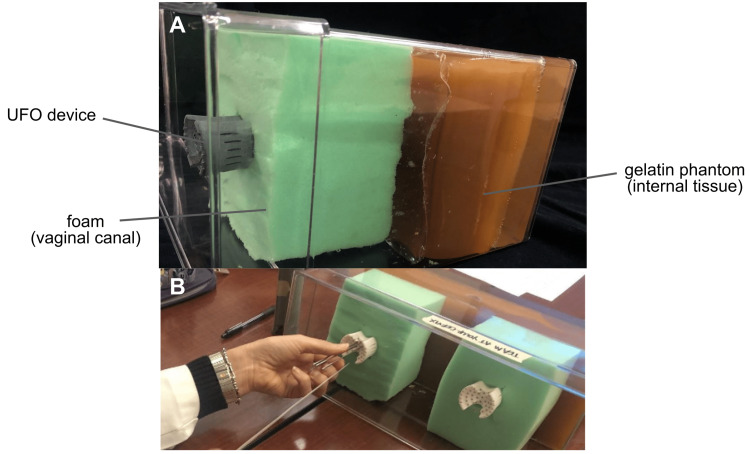
Testing for needle arrangement using a phantom (A) Testing system. (B) Needles being inserted into the prototype during testing. UFO: Universally Friendly Obturator.

The prepared phantom mimicked the cervical tissue, effectively mirroring a procedure of needle insertion. With the UFO inserted into the phantom, three radiation oncologists threaded needles through the UFO (Figure 7), from the user interface to the head. To map their trajectories, once the needles exited each channel four centimeters past the end of the head, the location of the needles was marked on a strip of clear tape attached to the casing surrounding the phantom. After all needles had been threaded and marked, the tape was removed and overlaid on top of the standard arrangement of a Syed-Neblett template. The distance between the marking and the desired location was measured to determine the accuracy of needle placement using the UFO. The results were analyzed by determining the average offset and any patterns that existed in the arrangement of the needles. Following this simulated procedure, the physicians were asked to complete a survey based on the System Usability Scale, a widely used assessment used to quantify responses to a new design (Appendix). These results were analyzed using the established scoring system for a SUS, and the average score of all users was calculated. For the UFO to qualify as “usable,” every survey must receive a score of 70 or above, considered a “good” score in the literature related to SUS testing [12]. The physicians were also verbally interviewed about their experience, and their responses were noted.

Testing for Needle Stability

**Figure 8:**
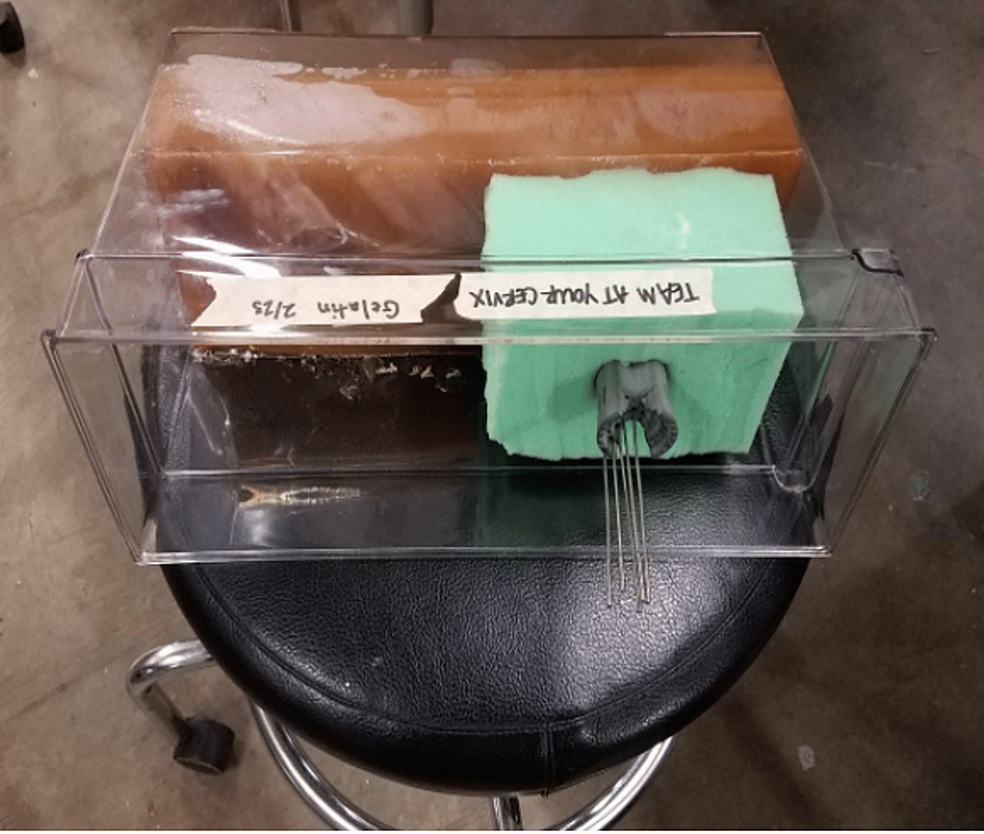
Testing for needle stability using a phantom

When patients undergo brachytherapy procedures, they are subject to significant movement, particularly when traveling from the operating room where the needles are placed to the clinic where the radiation is administered. To replicate this motion associated with treatment, the phantom, with the UFO inserted into it, was strapped to a stool, needles were inserted, and where each needle entered the UFO was marked (Figure 8). The stool was then pushed around multiple surfaces, like cement and cobblestone, and even carried inside an elevator to mimic the movement of a patient on a gurney. Calipers were used to measure the distance between the original position and the final position to determine if the needles were displaced. The test was considered successful if the displacement was less than or equal to 1 centimeter.

Cost Comparison

Taking standard applicators, specifically the Syed-Neblett as an example, a cost-comparison analysis of payer costs was conducted comparing the costs associated with the UFO to current comparable competitors. The costs were broken down into four categories: (1) personnel, (2) facility and equipment, (3) consumable supplies and sterilizing, and (4) procedural costs. Personnel, facility, and equipment costs were calculated by adjusting time-driven activity-based costing taken from prior brachytherapy cost literature to match the appropriate treatment regimens for each of the applicators [13]. Consumable supplies and sterilizing costs (Foley catheter, Omnipaque, clindamycin, prolene sutures, drapes, gloves, etc.) were found to be negligible between the treatment regimens, and thus a standardized cost was used across all regimens. Procedural costs were determined using the Current Procedural Terminology (CPT) coding system and the 2022 Medicare Physician Fee Schedule [14]. Costs were modeled using Medicare’s fee-for-service (FFS) pricing. Treatment regimens were assumed to be three fractions with three OR procedures with no overnight stays for the UFO applicators and four fractions with one OR procedure with two overnight stays for the Syed-Neblett applicator [15].

Results

Results of Usability and Needle Arrangement Testing

**Figure 9:**
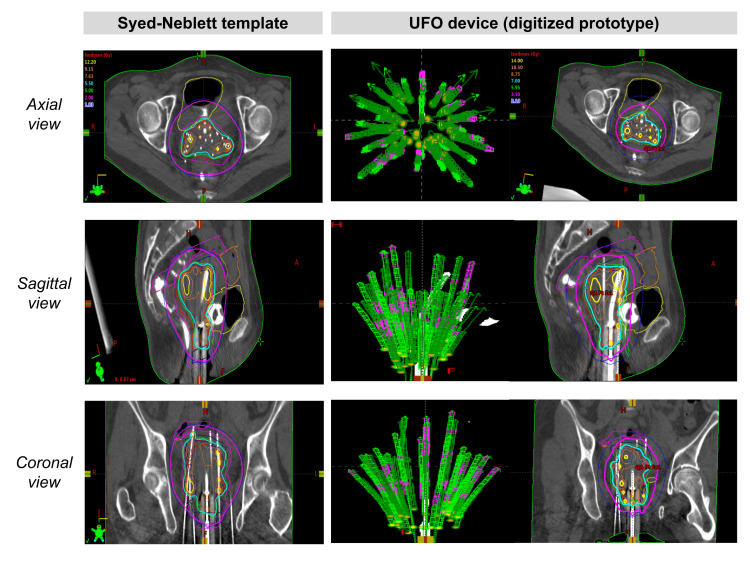
Comparing results of HDR planning software with Syed-Neblett template to UFO device HDR: high-dose rate (brachytherapy); UFO: Universally Friendly Obturator.

HDR planning software demonstrated minimal differences between the theoretical needle trajectories mapped using the standard Syed-Neblett template compared to a digitized version of the UFO (Figure 9). This outcome is reassuring, suggesting that the UFO can meet current expectations for transcutaneous needle placement even as an entirely vaginal applicator.

Usability testing resulted in an average SUS score of 80 from three experienced radiation oncologists, indicating acceptable usability of the UFO given that scores of 70 or above qualify a device as “usable.” Written comments from the SUS demonstrated that the UFO addressed concerns healthcare professionals experienced with brachytherapy have with current equipment and that the UFO would be easier for inexperienced healthcare professionals to learn to use compared to current equipment. Subsequent verbal interviews with the test subjects revealed that their estimated time for a procedure using the UFO would be 1.0 to 1.5 hours, a significant reduction given that the current procedure typically takes 2.0 to 2.5 hours for a skilled physician.

**Figure 10:**
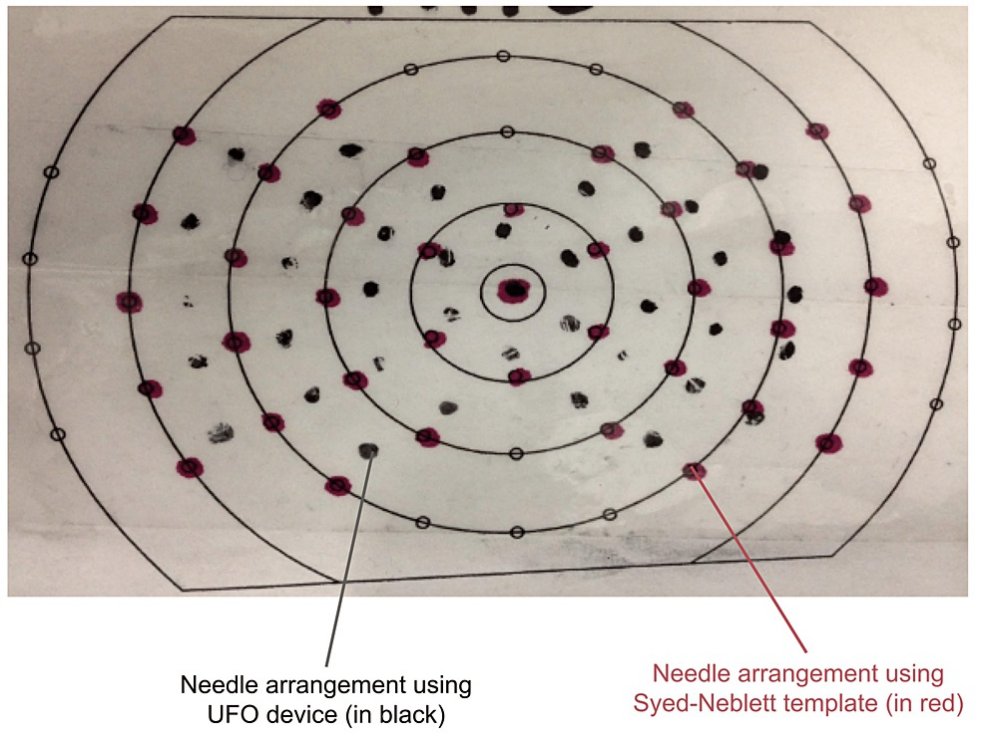
Comparing results of needle arrangement with Syed-Neblett template to UFO device UFO: Universally Friendly Obturator.

Needle arrangement testing resulted in an average offset of 5.5 millimeters from the Syed-Neblett template (Figure 10). While this offset measurement is significant, these results are likely caused by unanticipated changes in the needle movements through the simulated tissue due to issues with preparation and dimension. Moreover, especially when compared with results from HDR software modeling, this offset likely does not represent the performance of the UFO.

Results of Needle Stability Testing

After significant movement, there were zero discernible movements of the needles.

Cost Comparison

Using the prototype, it was calculated that the costs of implementing the UFO applicator ($16,786.09) were significantly less than the current costs of using the Syed-Neblett template ($47,418.77) overall [16,17]. Further cost savings arise from the manufacturing of the device itself, as Syed-Neblett templates can cost up to $30,000 based on obtained sales quotes. Though the manufacturing costs of the UFO may vary, the cost analysis still demonstrates that this design allows for a more cost-effective and flexible approach to brachytherapy compared to current standards (Table 1).

**Table 1:** Comparing costs of using the Syed-Neblett template to the Universally Friendly Obturator (UFO) device HDR: high-dose radiation; TPS: treatment planning system; T&O: tandem and obturator; E/M: evaluation and management.

	Syed-Neblett template			UFO device		
Personnel costs	Hourly pay	# of hours	Cost	Hourly pay	# of hours	Cost
Radiation oncology attending	320.40	3.00	961.20	320.40	3.00	961.20
Radiation oncology resident	30.60	4.00	122.40	30.60	4.00	122.40
Radiation oncology nurse	46.20	2.00	92.40	46.20	2.00	92.40
Medical physicist	110.40	2.00	220.80	110.40	2.00	220.80
Dosimetrist	63.60	2.00	127.20	63.60	2.00	127.20
Anesthesia attending	286.20	1.25	357.75	286.20	1.25	357.75
Anesthesia resident	30.60	1.25	38.25	30.60	1.25	38.25
Anesthesia nurse	120.00	1.25	150.00	120.00	1.25	150.00
Radiation therapist	45.00	1.00	45.00	45.00	1.00	45.00
Reception staff	18.00	1.00	18.00	18.00	1.00	18.00
Total personnel costs			2,133.00			2,133.00
Facility and equipment costs	Cost per treatment ($)	# of treatments	Total cost ($)	Cost per treatment ($)	# of treatments	Total cost ($)
Brachytherapy CT scanner	543.50	4.00	2,174.00	543.50	3.00	1,630.50
HDR afterloader	434.80	4.00	1,739.20	434.80	3.00	1,304.40
Brachytherapy TPS	614.15	4.00	2,456.60	614.15	3.00	1,842.45
Hospital inpatient stay	13,600.00	2.00	27,200.00	13,600.00	0.00	0.00
Total facility and equipment costs			33,569.80			4,777.35
Procedural costs	Technical costs ($)	Professional costs ($)	Total cost ($)	Technical costs ($)	Professional costs ($)	Total cost ($)
Clinical treatment planning						0.00
77263 Complex treatment planning	166.58	0.00	166.58	166.58	0.00	166.58
77470 Special treatment procedure	137.74	1.00	138.74	137.74	1.00	138.74
77316-77318 Brachytherapy isodose planning	1,230.25	4.00	1,234.25	1,301.20	3.00	1,304.20
Applicator placement						0.00
57155 T&O placement	1,434.45	0.00	1,434.45	0.00	0.00	0.00
55920 Needle placement	0.00	0.00	0.00	1,852.12	0.00	1,852.12
Simulation and imaging						0.00
77290 Complex simulation	2,344.80	4.00	2,348.80	1,875.84	3.00	1,878.84
76942 Ultrasound - needle placement	0.00	4.00	4.00	238.12	3.00	241.12
76965 Ultrasound - interstitial applicator	0.00	1.00	1.00	94.47	0.00	94.47
72197 MRI pelvis	0.00	1.00	1.00	369.59	1.00	370.59
77012 CT - needle placement	0.00	0.00	0.00	0.00	0.00	0.00
77021 MRI - needle placement	0.00	0.00	0.00	0.00	0.00	0.00
Dosimetry and physics						0.00
77332 Treatment devices	195.50	4.00	199.50	156.40	3.00	159.40
77336 Continuing physics consultation	420.45	0.00	420.45	336.36	0.00	336.36
77370 Special medical physics consultation	133.93	0.00	133.93	133.93	0.00	133.93
Treatment delivery						0.00
77770-77772 HDR treatment	3,026.35	4.00	3,030.35	2,421.08	3.00	2,424.08
C1717 Ir-192 HDR source	1,708.60	4.00	1,712.60	1,366.88	3.00	1,369.88
99152 Moderate sedation - initial	12.80	0.00	12.80	12.80	0.00	12.80
99153 Additional sedation	22.14	0.00	22.14	22.14	0.00	22.14
Observation care E/M						0.00
99218 - 99220 Observation care - initial	0.00	0.00	0.00	132.54	0.00	132.54
99224 - 99226 Observation care - subsequent	0.00	0.00	0.00	70.94	0.00	70.94
99217 Observation care - discharge	0.00	0.00	0.00	71.64	0.00	71.64
99234-99236 Observation - same day	0.00	0.00	0.00	0.00	0.00	0.00
Total procedural costs			10,860.59			10,780.37
Total cost			46,563.39			17,690.72

## Discussion

**Figure 11:**
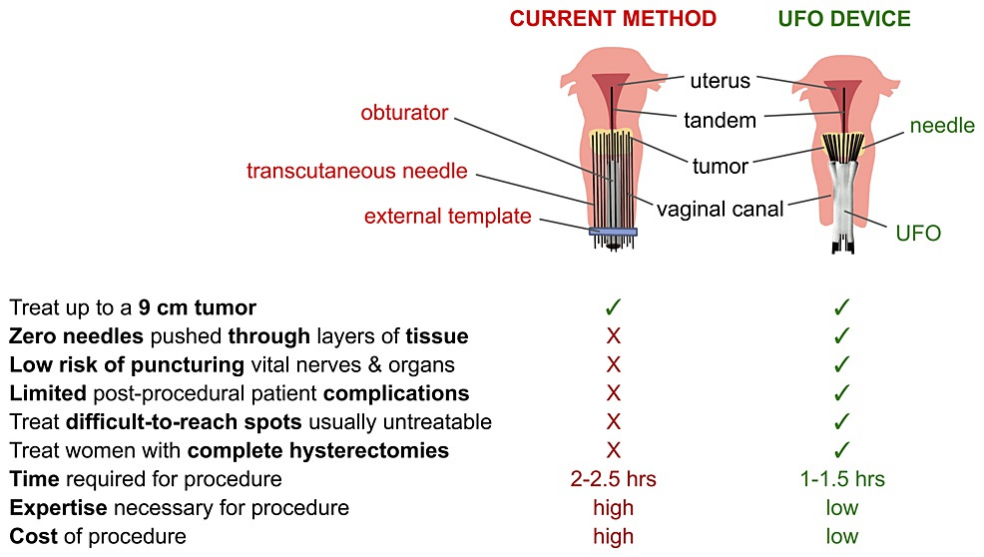
Comparing the use of the device to the current method of brachytherapy UFO: Universally Friendly Obturator; cm: centimeters; hrs: hours.

Given the success of preliminary testing of its current design, the UFO appears to exceed expectations by meeting its criteria to improve current brachytherapy methods for the treatment of cervical cancer (Figure 11). The device easily fits into current standards for the procedure by being compatible with most brachytherapy equipment, being compatible with CT imaging for dosimetric planning, and being available in multiple options for varying patient anatomy. With its angled channels serving as an internal template, the UFO is uniquely capable of treating up to a nine-centimeter (in transverse dimension) tumor with zero transcutaneous needles, sparing patients from the morbidity and post-procedural patient complications typically associated with brachytherapy procedures. And unlike external templates like the Syed-Neblett template, the channels also allow for precise control of needle arrangement at the level of the cervix. By ensuring exactly where each needle is going, healthcare professionals are more accurately able to plan dose distribution, potentially reducing the total number of needles needed to be placed for each patient. By simplifying needle placement, the device reduces the time, money, and expertise usually required for the procedure, addressing concerns associated with implementing brachytherapy in low-resource settings like LMICs.

Testing revealed that the UFO matches the Syed-Neblett template as the current standard of dose distribution, further demonstrating its efficacy in implementation. Planning also confirmed that by significantly reducing the likelihood of puncturing surrounding vital structures, the path of the angled channels also enables the UFO to guide needles to locations closer to these structures where tumors normally cannot be treated, improving the overall effectiveness of treatment. Moreover, with the adaptability of its central channel, UFO can also be optimized to treat patients with complete hysterectomies, unlike conventional methods that continue to fail to provide comprehensive radiation to these patients.

Future considerations

There are a number of limitations of the design that will need to be addressed in future iterations. Limitations of the UFO include determining how to upscale manufacturing; until now, the UFO has been fabricated using 3D printing to allow for rapid prototyping at a low cost. But to implement the UFO at a large scale globally, it will be pertinent to manufacture at a large scale not only to increase supply but also to lower the cost of each individual unit. Methods like injection molding may work to make this scaling possible. Moreover, to optimize the UFO for manufacturing, its material, and possibly its design, will need to change.

Another limitation of the design concerns sterilizability; the narrow channels of the UFO may be difficult to effectively clean, even given methods like steam sterilization. Therefore, further testing of the design is needed to determine its potential for sterilizability. Based on the manufacturing process and associated costs, another option may be to make the UFO single-use, eliminating the need for sterilization. However, it would be ideal for the UFO to be reusable, as the device should be able to be widely implemented in low-resource settings with financial limitations.

Currently, the UFO cannot be used to treat any lower vaginal involvement, since needles are guided to exit right at the cervix. Many late-stage cervical cancer cases can involve such metastases, and additional modifications can be explored that allow the UFO to also treat tumors in this region. With further development, research, and testing, all these considerations will be addressed in future designs.

Continued development

The UFO continues to undergo design updates, iterative testing, and adaptation for large-scale manufacturing by motivated engineers within the Global Medical Innovation program at Rice University. At the Baylor College of Medicine, motivated radiation oncologists continue to conduct preclinical testing on dosimetric planning, sterilization, and clinical implementation.

An intellectual property agreement has already been established, and a patent has been obtained with the U.S. Patent and Trademark Office (Serial No. 17/644,124, filed June 16, 2022). For regulatory compliance, the UFO should be classified as a Class II medical device qualifying for FDA Premarket Notification 510(k) because it appears to be substantially equivalent to brachytherapy applicators in the market [18]. Substantial equivalence is determined when a device has the same intended use and characteristics as its counterparts and does not pose any new safety risks. Assuming the UFO meets these requirements, the UFO should receive its FDA exemption within a year. Though legal protection and regulatory compliance are currently only being pursued in the United States, efforts to implement the UFO abroad are also being made, especially in LMICs with the greatest burden of late-stage cervical cancer, and the project continues to seek partnerships that will help on this journey.

Regarding the possible implementation of the UFO, once this progress has been made, several factors have been considered. LMICs may not have access to 3D-printing technology, but this is why we prioritized designing a device that, once produced, could simply be sent off for use and easily sterilized for re-use. Moreover, we used 3D-printing technology to develop the prototype as 3D-printing easily facilitates the design process, allowing us to make small changes with each iteration; so if other manufacturing techniques work better for mass-producing the UFO, then we would no longer need to rely on 3D-printing. Nevertheless, we would hope to produce the device ourselves, perhaps in the United States where we can ensure that certain regulatory standards are met, to be sent to facilities at LMICs for use.

We are confident and enthusiastic about the potential impact of the UFO, but we recognize that the continued progress of this project is dependent on the support of healthcare professionals around the world.

## Conclusions

We conclude that our novel device demonstrates the potential to safely and effectively improve upon current methods of brachytherapy in treating complex cases of cervical cancer. Moreover, our experience designing this device highlights that all healthcare professionals should not only feel capable of identifying concerns that may be addressed with technological solutions but also feel empowered to reach out to community partners, like local universities and engineering teams, for collaboration. Often, healthcare professionals may feel as though there may not be much they can do to change the status quo, especially when it comes to medical technology, but even if they lack certain technical abilities, their skills, like their experiences with patients and their understanding of procedures, are still instrumental in medical innovation.

## Appendices

**Table 2:** System Usability Scale (SUS) used to evaluate the device during testing UFO: Universally Friendly Obturator.

	Strongly disagree	Disagree	Neutral	Agree	Strongly agree
I felt very confident using the UFO.					
I thought the UFO was very easy to use.					
I think that the UFO would be successful in performing the procedure.					
I think that I would like to use the UFO frequently if I could.					
I think the UFO addresses my concerns with the current applicator.					
I found the UFO to be unnecessarily complex.					
I think that I would need extensive training to be able to use the UFO.					
I thought there were too many issues with the UFO to be used in its current state.					
I would imagine that most experienced healthcare professionals with prior experience performing cervical cancer brachytherapy would learn to use the UFO very quickly.					
I would imagine that most inexperienced healthcare professionals with no prior experience performing cervical cancer brachytherapy would learn to use the UFO very quickly.					
